# SISTER OF FCA physically associates with SKB1 to regulate flowering time in *Arabidopsis thaliana*

**DOI:** 10.1186/s12870-024-04887-y

**Published:** 2024-03-15

**Authors:** Chunhong Qiu, Tengyue Wang, Hui Wang, Zhen Tao, Chuanhong Wang, Jing Ma, Shuai Li, Yibing Zhao, Jifang Liu, Peijin Li

**Affiliations:** The National Key Engineering Lab of Crop Stress Resistance Breeding, Schoolof Life Sciences, Anhui Agricultural University, Hefei, 230036 China

**Keywords:** SKB1, SSF, *FLOWERING LOCUS C*, Flowering time, RNA-seq analysis

## Abstract

**Background:**

Proper flowering time is important for the growth and development of plants, and both too early and too late flowering impose strong negative influences on plant adaptation and seed yield. Thus, it is vitally important to study the mechanism underlying flowering time control in plants. In a previous study by the authors, genome-wide association analysis was used to screen the candidate gene *SISTER OF FCA* (*SSF*) that regulates *FLOWERING LOCUS C* (*FLC*), a central gene encoding a flowering suppressor in *Arabidopsis thaliana*.

**Results:**

SSF physically interacts with Protein arginine methyltransferase 5 (PRMT5, SKB1). Subcellular co—localization analysis showed that SSF and SKB1 interact in the nucleus. Genetically, *SSF* and *SKB1* exist in the same regulatory pathway that controls *FLC* expression. Furthermore, RNA-sequencing analysis showed that both *SSF* and *SKB1* regulate certain common pathways.

**Conclusions:**

This study shows that PRMT5 interacts with SSF, thus controlling *FLC* expression and facilitating flowering time control.

**Supplementary Information:**

The online version contains supplementary material available at 10.1186/s12870-024-04887-y.

## Background

In plants, flowering refers to the transition from vegetative to reproductive growth, representing an important turning point in the life cycle of plants. Appropriate flowering is crucial for plant adaptation and reproductive success. When to initiate flowering is determined by a combination of endogenous factors [1] and a multitude of external environmental cues [2–4]. Six different main pathways that control flowering have been identified: photoperiod, vernalization, autonomous, gibberellin, ambient temperature, and aging [5–7]. These pathways not only respond independently to flowering regulation signals, but also interact with other pathways to form a network that regulates flowering in a coordinated manner. *FLOWERING LOCUS C* (*FLC*), *FLOWERING LOCUS T* (*FT*), and *SUPPRESSOR OF OVEREXPRESSION* (*SOC1*) are regulated. They either activate or suppress the expression of downstream genes regulating inflorescence meristem and flower organs such as *LEAFY* (*LFY*), *APETALA1* (*AP1*), and *FRUITFULL* (*FUL*) to ensure that flowering is induced at the most appropriate time [8].

*FLC* encodes a MADS-box transcription factor and is an effective flowering repressor that inhibits flowering transition [9]. It has been reported that autonomous and vernalization pathways control flowering by repressing *FLC* expression [10]. The *SISTER OF FCA* (*SSF*) gene has been previously identified as a new autonomous pathway gene regulating *FLC*. *SSF* is a homologous gene of *FCA* that encodes a component of the autonomous pathway and an RNA processing factor containing a WW domain. *ssf* mutants exhibit early flowering and their *FLC* expression levels are reduced compared with the corresponding wild types under both long- and short-day growth conditions [11].

Numerous studies have shown that *FLC*-mediated flowering is associated with histone covalent modification, including methylation and acetylation [12]. For example, the vernalization response protein VERNALIZATION INSENSITIVE 3 (VIN3) can deacetylate histones at the *FLC* promoter region, thus silencing *FLC* repression during vernalization treatment; VERNALIZATION1 (VRN1) and VERNALIZATION2 (VRN2) mediate methylation modification of H3K9 and H3K27 sites, thus reducing *FLC* repression and inducing early flowering [13]. In addition to the methylation of lysine, the histones in arginine can also be modified by methylation. Methylation of arginine histones mainly happens at the arginine 2 (R2), 8 (R8), 17 (R17), and 26 (R26) sites of histone H3 or at the arginine 3 (R3) site of histone H4 [14]. Arginine can be either symmetrically or asymmetrically methylated, depending on the number of methylated guanidine groups [15]. The arginine methylation modification is mediated by a set of evolutionarily conserved arginine methyltransferases (PRMTs) [16]. *Arabidopsis thaliana* (*Arabidopsis*) has nine homologous proteins of PRMT (i.e., AtPRMT1a, AtPRMT1b, AtPRMT3, AtPRMT4a, AtPRMT4b, AtPRMT5, AtPRMT6, AtPRMT7, and AtPRMT10), seven of which have been functionally reported to date [17]. Several studies have shown that PRMT4a/4b, PRMT5, and PRMT10 mediate arginine histone methylation, thus regulating flowering time by repressing *FLC* expression [18–21].

AtPRMT5, which is also designated SKB1, is a type II arginine methyltransferase in *Arabidopsis* that catalyzes the H4R3 symmetric dimethylation of chromatin in the *FLC* promoter region, thus repressing *FLC* expression and promoting flowering. Compared with wild-type plants, *Arabidopsis skb1* mutant plants exhibit late flowering, darker leaf color, and a slower growth rate [22]. Zhang et al. found that *prmt5/skb1* mutants are salt intolerant, which was associated with reduced arginine methylation of small nuclear ribonucleoprotein Sm-like4 (LSM4) and impaired pre-mRNA splicing of stress-related genes [23]. *PRMT5/SKB1* is also required to maintain *Arabidopsis* root stem cells in response to DNA damage [24]. In addition, Hu et al. reported that nitric oxide positively regulates the methyltransferase activity of *Arabidopsis* PRMT5 through s-nitrite acylation of Cys-125 during the stress response. This acylation results in the correct splicing of stress-related gene-specific precursor mRNA, ultimately improving stress tolerance [25]. Recent studies have shown that *AtPRMT5* is also involved in plant immunity. Bacterial infection leads to downregulation of *PRMT5* expression, resulting in reduced arginine methylation of AGO2 and promoted plant immunity. Consistent with this result, the *atprmt5* mutant showed increased resistance to bacteria [26].

In this study, the partners of SSF were examined using immunoprecipitation-mass spectrometry (IP-MS) and the candidate interaction protein SKB1 was identified. SSF and SKB1 were found to interact in the nucleus. Furthermore, SSF and SKB1 are involved in the same genetic pathway that regulates *FLC* and flowering time. RNA-sequencing (RNA-Seq) analysis showed that compared with wild-type plants, 44 common differentially expressed genes (DEGs) were identified in *ssf-2* and *skb1-1*. These experimental findings expand the role SSF plays in flowering time regulation, which is useful for the mining of new genes for the regulation of plant flowering time.

## Methods

### Plant materials and growth conditions

All *Arabidopsis* lines used in this study have a Col-0 background. *ssf-2* and *skb1-1* mutants were ordered from the Nottingham *Arabidopsis* Stock Centre, and have been used in previous studies. Homozygous mutants were identified by PCR using genotyping primers from the TAIR website. *Arabidopsis* seeds were surface sterilized with chlorine gas and then plated on half-strength Murashige and Skoog (½ MS) medium. After being incubated in the dark at 4 ℃ for 3 d, plates were transferred to long-day growth conditions (16 h light/8 h dark) at 22 ℃. After two weeks, the seedlings were transplanted to soil for additional growth under the same conditions. The total leaf number was used as an indicator of the flowering time. Primer sequences for PCR genotyping are presented in Table S1.

### Yeast two hybrid assay (Y2H)

The coding sequences (CDS) of *SSF* and *SKB1* were amplified with gene-specific primers, and PCR products were inserted into pGBKT7 and pGADT7 in-frame with their binding domain (BD) and activation domain (AD) sequences, respectively. Both pGBKT7-SSF and pGADT7-SKB1 were co-transformed into the yeast strain AH109 following the Yeast Protocols Handbook (Clontech). Mated clones were selected on SD/-Leu/-Trp medium, whereas interactions were selected on SD/-Leu/-Trp/-His/-Ade (SD-LWHA) medium. Yeast plates were incubated for up to 4 d at 30 ℃ before being photographed. Primer sequences for vector construction are presented in Table S1.

### Bimolecular fluorescence complementation assay (BiFC)

The CDS of *SSF* and *SKB1* were cloned into pSPYNE-YFP and pSPYCE-YFP vectors in-frame with NE-YFP and CE-YFP, respectively. pSPYNE-SSF-YFP and pSPYCE-SKB1-YFP were co-transformed into *A. thaliana* protoplasts. pSPYCE-SKB1-YFP + pSPYNE-YFP and pSPYNE-SSF-YFP + pSPYCE-YFP were used as negative controls. YFP fluorescence was observed 2 d after the transformation using a confocal microscope (Carl Zeiss LSM800). Primer sequences for these constructs are shown in Table S1.

### Luciferase complementation assay (LCI)

The CDS of *SSF* and *SKB1* were cloned into 772-cLUC and 771-nLUC vectors in-frame with the C and N terminal halves of LUC, respectively. 772-cLUC-SSF and 771-nLUC-SKB1 were introduced into the *Agrobacterium* strain GV3101. GV3101 strains with LCI constructs were injected into *Nicotiana benthamiana* leaves (using 772-cLUC + 771-nLUC, 772-cLUC-SSF + 771-Nluc and 771-nLUC-SKB1 + 772-cLUC as negative controls). After injected plants had grown for 48 h, the reaction substrate fluorescein was sprayed onto leaf surfaces, and fluorescence signals were detected using a luminometer (Chemiluminescence image analysis system, Tanon, China). The primer sequences for these constructs are presented in Table S1.

### Construction of *SKB1-amiR*

An artificial microRNA (amiR) targeting SKB1 was designed as previously described [27]; it was amplified using plasmid pRS300 as template and then ligated into a modified version of the pCambia1300m vector. All primer sequences for these constructs are presented in Table S1.

### RNA-seq analysis

Total RNA was extracted from two-week-old seedlings of wild-type Col-0, *ssf-2*, and s*kb1-1* mutants grown on ½ MS medium under long-day (16 h light/8 h dark) conditions using the Trizol reagent (Invitrogen, CA, USA) following the manufacturer's protocol. RNA‐seq was conducted by Lianchuan Biotechnology Company (Hangzhou, China) using three biological replicates. A cDNA library, constructed from the pooled RNA of the three samples, was sequenced with the Illumina 4000 sequence platform. The mapped reads of each sample were assembled using StringTie. StringTie was also used to assess the expression levels of mRNAs by calculating the fragments per kilobase of feature per million mapped reads (FPKM). The DEGs were identified with |log2 (fold change) |≥ 1 and *p* value < 0.05 by the R package. The Kyoto Encyclopedia of Genes and Genomes (KEGG) source was obtained from the KEGG database [28]. KEGG pathway enrichment analysis of DEGs was performed using the KOBAS2.0 wbsite (http://kobas.cbi.pku.edu.cn/home.do). The transcript level was calculated based on FPKM. log2 (FPKM + 1) values were used to analyze gene expression. The raw sequence data have been submitted to the NCBI Short Read Archive under accession number PRJNA1037613.

### RNA extraction and real time–quantitative polymerase chain reaction real-time (RT-qPCR) analysis

All gene expression analyses conducted in this study were performed using two-week-old seedlings grown under long-day (16 h light/8 h dark) growth conditions at 22 ℃. Total RNA was extracted with the hot phenol extraction method [29]. Reverse transcription was conducted using the Invitrogen Superscript III Reverse Transcription System following the manufacturer’s protocol. RT-qPCR was performed on a Roche 480II machine with gene-specific primers. Primer sequences are shown in Supplementary Excel 1. The *UBIQUITIN C* (*UBC*) gene was used as an internal control for RT**-**qPCR analysis. The 2^−ΔCt^ method was used to calculate the normalized expression of target genes.

### Chromatin immunoprecipitation (ChIP) assays

Histone ChIP assays were performed as previously described [11, 30–32]. Briefly, 3 g of 14-day-old seedlings was fixed with 1% (v/v) formaldehyde. Nuclei were extracted and sheared to an average DNA fragment size of approximately 200–500 bp by sonication. After chromatin had been precleared with protein A/G magnetic beads, protein-DNA complexes were precipitated with anti-Histone H4 Antibody (Millipore; 05–858), and anti-Histone H4 (symmetric di methyl R3)-ChIP Grade (ab5823, Abcam). The protein-DNA complexes were reverse cross-linked overnight with 0.2 M NaCl at 65 °C. The precipitated DNA fragments were recovered and analyzed by qPCR using specific primers at different *FLC* genomic regions. The relative enrichments of various regions of *FLC* were calculated by normalizing H4Rme3 qPCR data to H4 qPCR data. Each sample was repeated three times. qPCR primer sequences are shown in Table S1.

### Statistical analysis

SPSS16.0 software was used to analyze the significance of the data using the default parameters settings. A two tailed unpaired *t*-test was applied to compare the data between two sample groups (*p < 0.05; **p < 0.01; ***p < 0.001; ns, no significant difference).

## Results

### SSF physically interacts with SKB1 in the nucleus

SSF regulates *FLC* transcription and flowering time in *Arabidopsis* by affecting RNA polymerase II enrichment at the *FLC* genomic region [11, 31]; however, the exact mechanism underlying the regulation of *FLC* transcription by SSF requires further research. To further dissect the molecular mechanism of SSF function, its protein interaction partners were screened by protein immunoprecipitation assays using GFP antibody and *ssf-2 proSSF:SSF-GFP* transgenic plants [11], followed by mass spectrometry analysis. According to the IP-MS results, all SSF-GFP transgenic plants were able to pull down the SKB1 protein, ranging from 8 to 19 peptides, whereas SKB1 peptides were not pulled down in Col-0 control samples (Table S2). Therefore, SKB1 could interact with SSF and further experiments focused on SKB1.

**Fig. 1:**
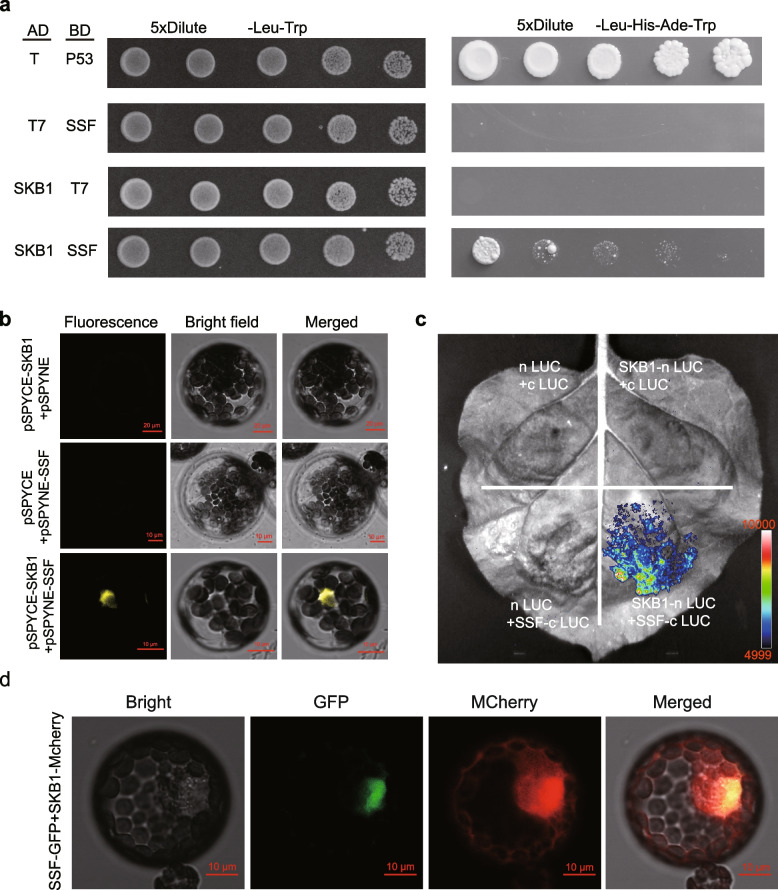
SSF interacts with SKB1 in nucleus. **a** SSF interacts with SKB1 in yeast cells. The interaction between T and P53 was used as positive control; pGBKT7 with SSF and SKB1 with pGADT7 were used negative control. **b**, **c** BiFC experiments using YFP (**b**) and split Luciferase (**c**) show that SSF interacts with SKB1 in *Arabidopsis* protoplast (**b**) and in *N. benthamiana* (**c**). **d** Co-localization of SSF and SKB1 in *Arabidopsis* protoplasts. Scale bar, 10 μm. All these experiments were repeated at least three times and the consistent results were shown in the figure

To validate the interaction between SKB1 and SSF, Y2H analysis was carried out first. The coding sequence of *SSF* was fused in frame with the binding domain in the pGBKT7 vector (BK-SSF) and *SKB1* with the activation domain in the pGADT7 vector (AD-SKB1). Compared with the negative controls including AD-SKB1 with T7 or T7 with BK-SSF, yeast cells containing pGBKT7-SSF and pGADT7-SKB1 constructs grew on SD medium devoid of leucine, histidine, adenine, and tryptophan (Fig. 1a). Next, a BiFC assay was performed to verify interactions. As shown in Fig. 1b, when SKB1-cYFP and SSF-nYFP were transiently co-expressed in *Arabidopsis* mesophyll protoplasts, a strong YFP signal was observed in the nucleus. However, co-expression of SKB1-cYFP or SSF-nYFP with empty vector did not lead to any visible fluorescence. Furthermore, an LCI assay was performed in *N. benthamiana* leaves. *N. benthamiana* leaves were co-infiltrated with constructs encoding SKB1-nLUC (SKB1 fused to the N-terminal of LUC), and cLUC-SSF (SSF fused to the C-terminal of LUC). Strong luciferase activity was observed when SKB1-nLUC and SSF-cLUC were co-expressed (Fig. 1c). Negative control assays containing nLUC with cLUC, SKB1-nLUC with cLUC, or nLUC with SSF-cLUC showed no fluorescence signals, further supporting the interaction of SSF and SKB1.

To identify the subcellular location where SSF and SKB1 interact, the subcellular localization of SKB1-GFP in the roots of *35S:SKB1-GFP* transgenic *Arabidopsis* seedlings was compared with SSF-GFP and SKB1-mCherry in the *Arabidopsis* protoplast. The results showed that SKB1 localized in the nucleus and cytosol while SSF localized predominantly in the nucleus (Supplementary Fig. 1). The merged fluorescence of the two proteins indicated that SKB1 and SSF co-localized in the nucleus (Fig. 1d). Taken together, these results demonstrate that SKB1 physically interacts with SSF in the *Arabidopsis* nucleus.

### *AmiR-SKB1* exhibits a late flowering phenotype

To confirm the biological function of SKB1 in flowering time regulation, a T-DNA insertion mutant *skb1-1* (SALK_065814) that has been reported by Wang et al. (2007) was analyzed [22]. Consistent with the previous finding, the *skb1-1* mutant appeared to be late flowering compared with Col-0. RT-qPCR was performed to analyze the expression level of unspliced (nascent) *FLC* and spliced (mature) *FLC* transcripts. Both transcripts showed significantly higher levels in the *skb1-1* mutant compared to wild-type Col-0 (Supplementary Figs. 2 and 3).

**Fig. 2:**
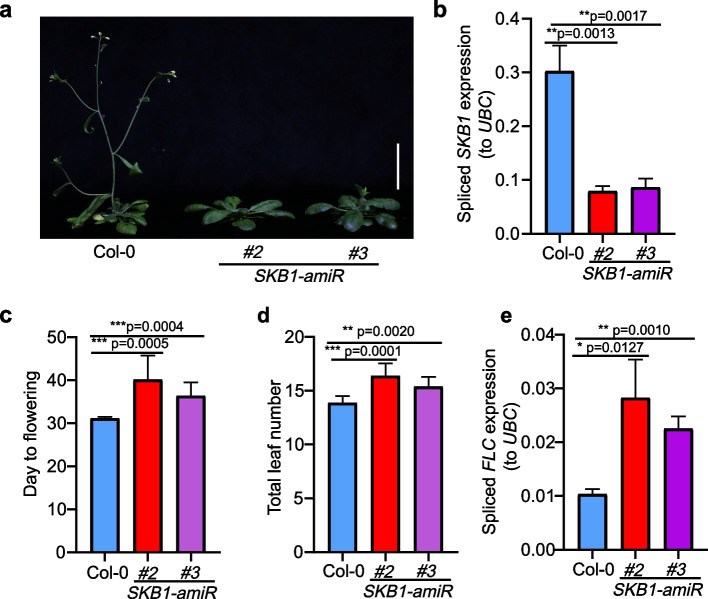
SKB1-amiR exhibits a late flowering phenotype. **a** The phenotype of *SKB1-amiR #2* and *#3*. **b** The expression of *SKB1* were down-regulated in *SKB1-amiR #2* and *#3* transgenic plants compared with Col-0 under long-day growth conditions. **c** The total leaf number of *SKB1-amiR #2* and *#3* (*n* = 8). **d** Day to flowering of *SKB1-amiR #2* and *#3*. **e** Spliced *FLC* expression were up-regulated in *SKB1-amiR #2* and *#3* transgenic plants compared with Col-0 under long-day growth conditions (*n* = 3)

To further verify the function of SKB1, additional transgenic SKB1-knockdown *Arabidopsis* lines were obtained, containing artificial microRNA targeting *SKB1* mRNA. The homozygous *SKB1-amiR* #2 and *SKB1-amiR* #3 exhibited a late flowering phenotype compared to the wild-type Col-0 (Fig. 2a), and the expression level of *SKB1* was significantly downregulated in both lines (Fig. 2b). Further quantitative analysis of the total number of leaves and days to flowering confirmed the flowering enhancing role of SKB1 (Fig. 2c, d). Moreover, the total RNA of these *SKB1-amiR* and Col-0 seedlings was extracted at the four-leaf stage and *FLC* expression analysis was performed. The results showed that the transcript levels of *FLC* of *SKB1-amiR* lines were significantly higher than that of Col-0 (Fig. 2e). Together, these results demonstrate that SKB1 promotes flowering by negatively regulating *FLC* expression.

### SSF and SKB1 are involved in mediating flowering gene expression

**Fig. 3:**
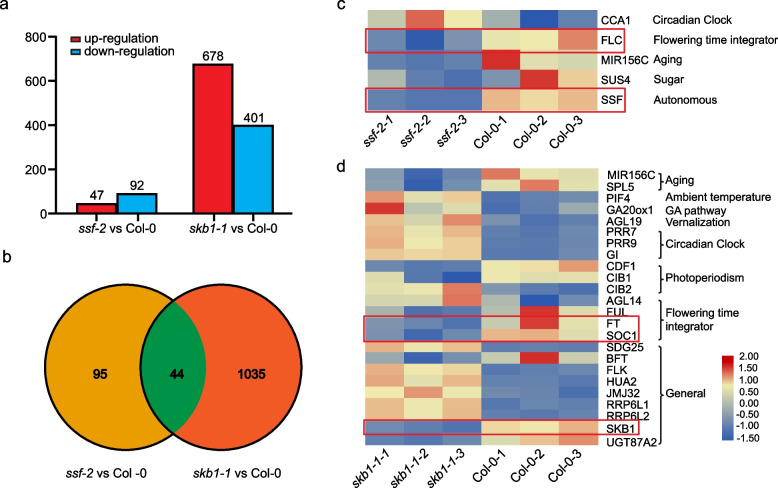
Analysis of differentially expressed genes in *ssf-2* and *skb1-1* compared with Col-0. **a** Overall analysis of significantly differentially expressed genes. **b** Analysis of common differentially expressed genes in *ssf-2* and *skb1-1* relative to Col-0. **c**, **d** Heatmap analysis of significant flowering related genes in *ssf-2* vs Col-0 (**c**) and *skb1-1* vs Col-0 (**d**)

To examine the molecular mechanism of how SKB1 and SSF regulate flowering time, an RNA‐seq analysis was performed on 14-day-old seedlings of Col-0, *skb1-1*, and *ssf-2* mutants. In *ssf-2* vs Col-0, 139 significant DEGs were identified: 47 were upregulated and 92 were downregulated (Fig. 3a). A total of 1,079 DEGs were identified in *skb1-1* vs Col-0, of which 678 were significantly upregulated and 401 were significantly downregulated (Fig. 3a). *ssf* and *skb1* shared 44 common DEGs (Fig. 3b). We further performed KEGG enrichment analysis on these 44 common genes and the results are shown in Supplementary Fig. 4: the protein processing in endoplasmic reticulum (ko04141), glutathione metabolism (ko00480), starch and sucrose metabolism (ko00500), MAPK signaling pathway –plant (ko04016) and plant hormone signal transduction (ko04075) pathways were enriched, which suggest that SSF and SKB1 may functions as diverse flowering modulators by regulating multiple pathways. Among the identified five flowering genes, *FLC* and miR156C were significantly downregulated in the *ssf-2* mutant, which is consistent with the results of the early flowering phenotype of *ssf-2* (Fig. 3c). Among the 1,079 DEGs from *skb1-1* vs Col-0, 24 flowering-related genes were found. The floral integration genes *FUL*, *FT*, and *SOC1* were significantly downregulated in the *skb1-1* mutant compared to Col-0. This result is consistent with the late flowering phenotype of *skb1-1* (Fig. 3d). Some of these genes were selected for further validation with a RT-qPCR method. The results showed that RT-qPCR and RNA-seq data were consistent with each other, thus supporting the reliability of RNA-seq analysis (Supplementary Fig. 5).

**Fig. 4:**
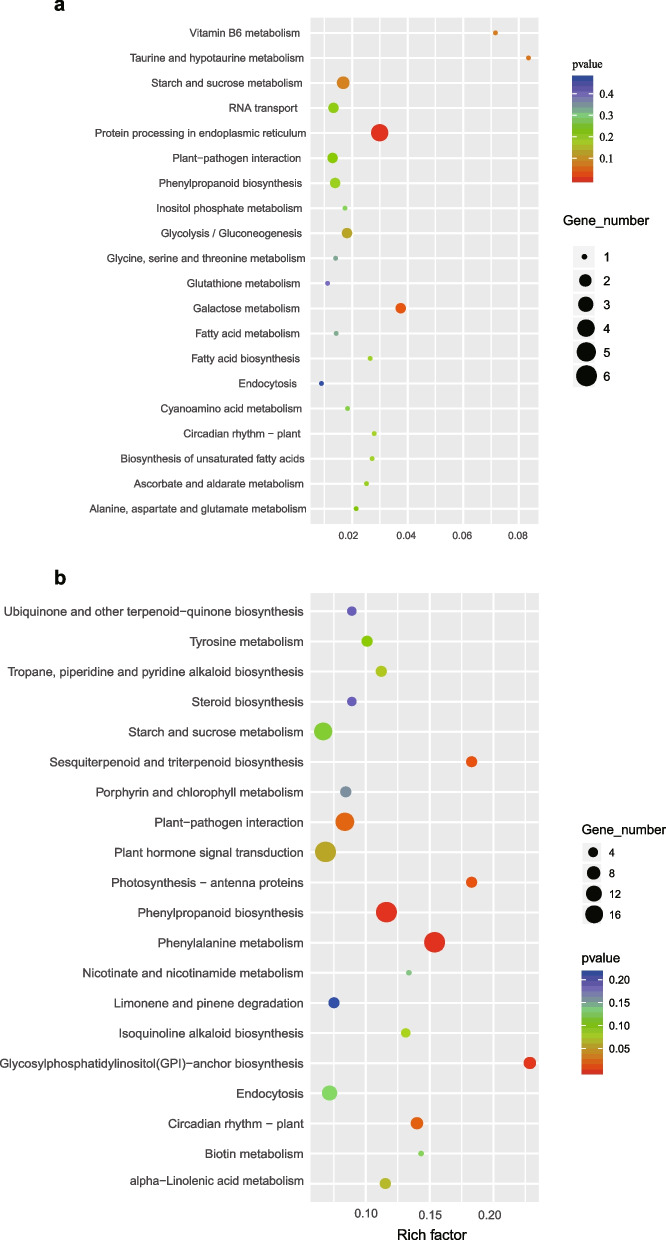
KEGG enrichment analysis of differentially expressed genes (DEGs) in *ssf-2* and *skb1-1* compared with Col-0. **a** KEGG enrichment analysis of DEGs in *ssf-2* vs Col-0. **b** KEGG enrichment analysis of DEGs in *skb1-1* vs Col-0

To further determine the metabolic pathways in the flowering process, KEGG enrichment analysis was performed. Circadian rhythms plant (ko04712) and starch and sucrose metabolism (ko00500) were significantly enriched in both *ssf-2* vs Col-0 and *skb1-1* vs Col-0 (Fig. 4a-b). In addition, a mapman pathway analysis was also conducted based on the log2FC of DEGs between *skb1-1* vs Col-0 and *ssf-2* vs Col-0 (Supplementary Fig. 6). These results suggest that SSF and SKB1 function as diverse flowering modulators by regulating multiple genetic pathways.

### SKB1 regulates *FLC* regulation independent of SSF

**Fig. 5:**
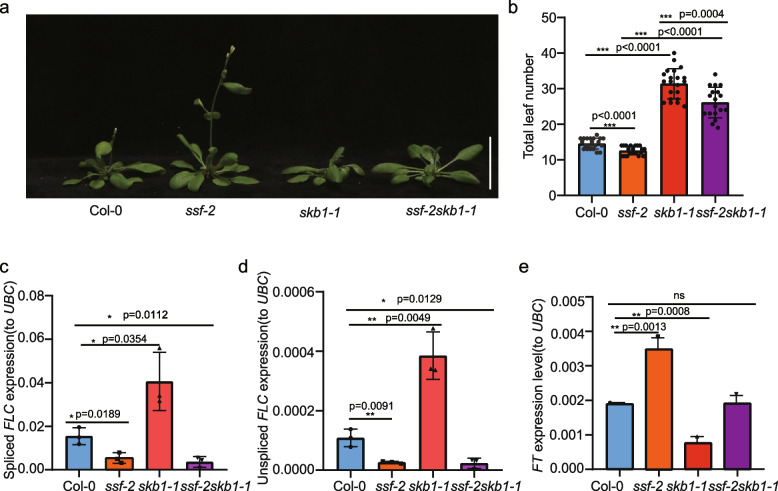
SKB1 downregulate *FLC* expression depend on SSF. **a**, **b** Flowering phenotype and leaf number of *ssf-2*, *skb1-1* and *ssf-2 skb1-1* (*n* = 12). **c**, **d** Expression level of spliced *FLC* and unspliced *FLC* in *ssf-2*, *skb1-1* and *ssf-2 skb1-1* (*n* = 3). e. Expression level of *FT* in *ssf-2*, *skb1-1* and *ssf-2 skb1-1*(*n* = 3). In (**b**), data are means ± SEM of 12 plants. In (**c**-**d**), data are means ± SEM of three replicated plates of 14-days-old seedlings. Significant differences were determined using Student’s *t*-test

Considering that *FLC* is a central component for flowering time control and is regulated by both SSF and SKB1, subsequent experiments focused on *FLC*. To examine the genetic relationship between SSF and SKB1 in the regulation of flowering time, *skb1-1* was crossed with *ssf-2* to produce the homozygous double mutant *ssf-2 skb1-1*. The *ssf-2 skb1-1* double mutant flowered later than *ssf-2* and earlier than *skb1-1* (Fig. 5a); consistently, the total leaf number of *ssf-2 skb1-1* was significantly higher than that of *ssf-2*, but lower than that of *skb1-1* (Fig. 5b). The *FLC* expression level was further examined in Col-0, *ssf-2*, *skb1-1*, and *ssf-2 skb1-1* mutants. Compared with Col-0, both unspliced *FLC* and spliced *FLC* expression levels significantly decreased in *ssf* and increased in *skb1-1*. Notably, the *FLC* expression of *ssf-2 skb1-1* was similar to that in *ssf-2* (Fig. 5c, d). These results suggest that *SSF* and *SKB1* act within the same genetic pathway in regulating *FLC* expression.

The *ssf-2 skb1-1* double mutant flowers far later than *ssf-2*, but the *FLC* expression level of *ssf-2 skb1-1* is similar to that of *ssf-2*. These two results are inconsistent given that *FLC* has been well-studied and identified as a potent flowering repressor [33]. Therefore, the expression of *FLOWERING LOCUS T* (*FT*)—the key florigen factor for flowering time regulation [34], which is repressed by *FLC*—was further examined [35]. The expression level of *FT* was significantly increased in *ssf-2* and significantly decreased in *skb1-1* mutant, and the *FT* expression of *ssf-2 skb1-1* was similar to that of Col-0. This result is consistent with the flowering time phenotype but differs from the *FLC* expression result (Fig. 5e). Therefore, it was hypothesized that in addition to affecting *FLC* via SSF, *SKB1* may also regulate *FT* and thus affect the flowering time through SSF independent pathways.

### SSF does not affect the enrichment of H4R3sme2 in the *FLC* region

**Fig. 6:**
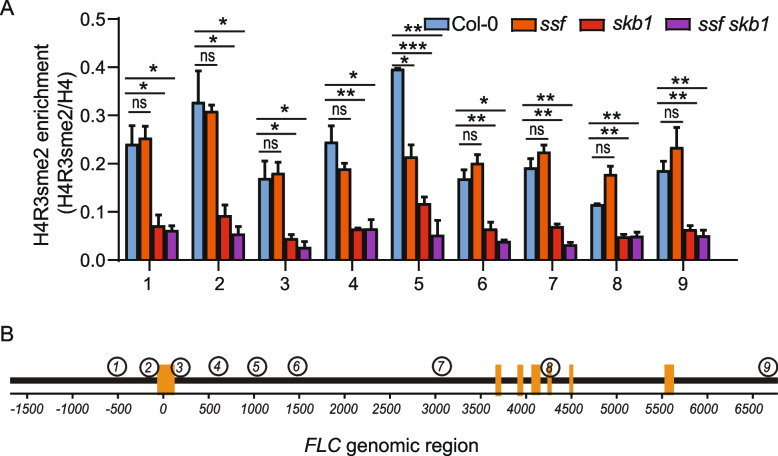
*FLC* H4R3sme2 ChIP in Col-0, *ssf-2*, *skb1-1* and *ssf-2 skb1-1*. **a** Accumulation of H4R3sme2 normalized to H4 in different regions of *FLC* (*n* = 3). **b** Schematic presentation showing the position of the qPCR primers in (**a**) in *FLC* genomic regions. In (**a**) data are presented as mean ± SEM. Asterisks indicate significant differences (*p < 0.05; **p < 0.01; ***p < 0.001, ns, not significant; two-tailed unpaired Student’s *t* test)

*SKB1* was reported to be involved in the symmetric dimethylation of histone H4R3 and in the control of the flowering time in *Arabidopsis* [22]. Therefore, the enrichment of H4R3sme2 at the *FLC* genomic region was examined using two-week-old seedlings of Col-0, *ssf-2*, *skb1-1*, and *ssf-2skb1-1*. Compared with wild-type Col-0, H4R3sme2 enrichment on *FLC* was significantly decreased in both *skb1-1* and *ssf-2skb1-1* mutants. However, no significant difference was found between *ssf-2* and Col-0, suggesting that SSF may regulate *FLC* expression through an H4R3sme2-independent mechanism (Fig. 6).

## Discussion

Flowering is a vitally important trait and more than 300 genes related to flowering regulation have been reported in *Arabidopsis* [36]. *FCA* and *FPA* have been identified to play a flowering regulatory role classified within the autonomous flowering pathway [37, 38]. Another example of an autonomous pathway is the identification of *SSF*. This gene regulates *FLC* and is a homolog of *FCA*, but its function for flowering time control is antagonistic to that of *FCA*, and *ssf* mutants exhibit early flowering [11].

In this study, through protein IP-MS, Y2H, BiFC and LCI analyses, it was confirmed that SSF physically interacts with SKB1 (Fig. 1). SKB1 is an arginine methyltransferase that regulates H4R3 methylation [22]. Compared with Col-0, *ssf-2* is early flowering and *skb1* is late flowering [11, 22], and the phenotype of *skb1* was further confirmed by artificial microRNA transgene analysis (Fig. 2). The genetic relationship between SSF and SKB1 was then determined by the double mutant *ssf-2 skb1-1*, which was obtained from a cross between *ssf-2* and *skb1-1* mutants. Phenotypic and leaf number analysis showed that the flowering time of *ssf-2 skb1-1* was intermediate (later than *ssf-2* and earlier than *skb1-1*), which was further supported by *FT* expression results (Fig. 5a, b, and e). However, while the *FLC* expression level of *ssf-2 skb1-1* was similar to that of *ssf-2*, it was significantly lower than that of *skb1-1* (Fig. 5c, d). This result supports the notion that SSF may play an important role in SKB1-regulated *FLC* expression*.* In a previous study, PRMT6 was proposed to be not only involved in the regulation of photoperiodic *FT* expression through the Nuclear Factor-CONSTANS module, but also that it exhibits redundancy with PRMT4a/PRMT4b to regulate *FLC* expression and promote floral transition in *Arabidopsis* [39]. The authors speculate that for flowering time regulation, in addition to affecting *FLC* via SSF, SKB1 may also regulate *FT* and thus affect flowering time through SSF independent pathways. In support of this speculation, KEGG enrichment analysis of *ssf-2* and *skb1-1* showed that SSF and SKB1 were involved in diverse metabolic pathways, such as circadian rhythms, hormone signal transduction, and starch-sucrose metabolism pathways (Fig. 4). This involvement suggests that these two genes may regulate flowering in multiple ways.

Arginine residue methylation is a widespread and relatively conserved post-translational modification of proteins in eukaryotes [14]. Arginine residue is methylated by a protein class called protein arginine methyltransferase (PRMT) protein family, which can methylate histones and a variety of non-histone proteins (including RNA-binding proteins) [15, 40]. PRMT participates in a variety of developmental processes and stress regulation by regulating the RNA post-transcription level. PRMT5 is the well-studied type II protein arginine methyltransferase, which catalyzes the formation of symmetric arginine dimethylation [41]. It has been reported that *SKB1* can catalyze H4R3sme2 to regulate flowering [22]. To explore the mechanism of how SSF and SKB1 regulate flowering, arginine methylation H4R3sme2 ChIP analysis was performed for the *FLC* genomic region. The results showed that SKB1 catalyzed enrichment of H4R3sme2 in the *FLC* region (Fig. 6). The *FLC* expression of *ssf-2 skb1-1* was similar to that in *ssf-2*, which suggests that SKB1 regulates *FLC* regulation in dependence on SSF. We speculated that SKB1 may repress SSF-mediated *FLC* expression through attenuating SSF association with *FLC* promoter region through H4R3sme2 modification. However, the exact mechanism of how the SSF-SKB1 regulates flowering needs to be further explored.

## Conclusions

In conclusion, in this study, the interaction between SSF and SKB1, the relationship between *SSF* and *SKB1* in flowering time regulation was uncovered, and DEGs and pathways regulated by *SSF* and *SKB1* were obtained. These results extend the knowledge on the function of *SSF* in flowering time control.

### Supplementary Information

**Supplementary Material 1.**

**Supplementary Material 2.**

**Supplementary Material 3.**

**Supplementary Material 4.**

**Supplementary Material 5.**

**Supplementary Material 6.**

**Supplementary Material 7.**

**Supplementary Material 8.**

## Data Availability

All data supporting the findings of this study have been included in the paper and are available from the corresponding author (PL) upon request. The raw sequence data have been submitted to the NCBI Short Read Archive (SRA) with accession number PRJNA1037613.
