# Supplementary figures and images for: SISTER OF FCA physically associates with SKB1 to regulate flowering time in Arabidopsis thaliana
Source: BMC Plant Biol. 2024 Mar 15;24:188. doi: 10.1186/s12870-024-04887-y (PMC10941358; doi:10.1186/s12870-024-04887-y)

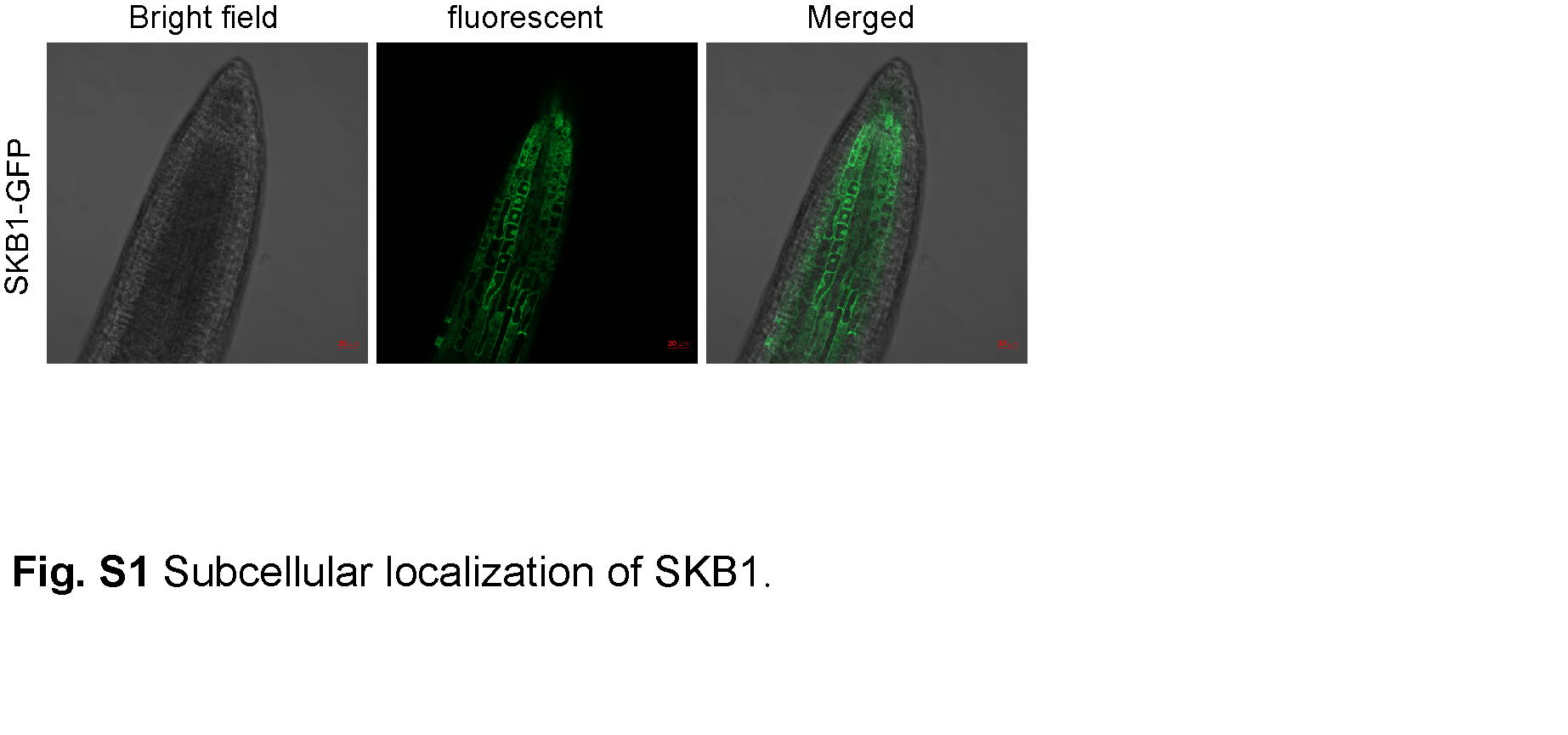

Supplement: Supplementary file 3 — Supplementary Material 3. [file 12870_2024_4887_MOESM3_ESM.tif]

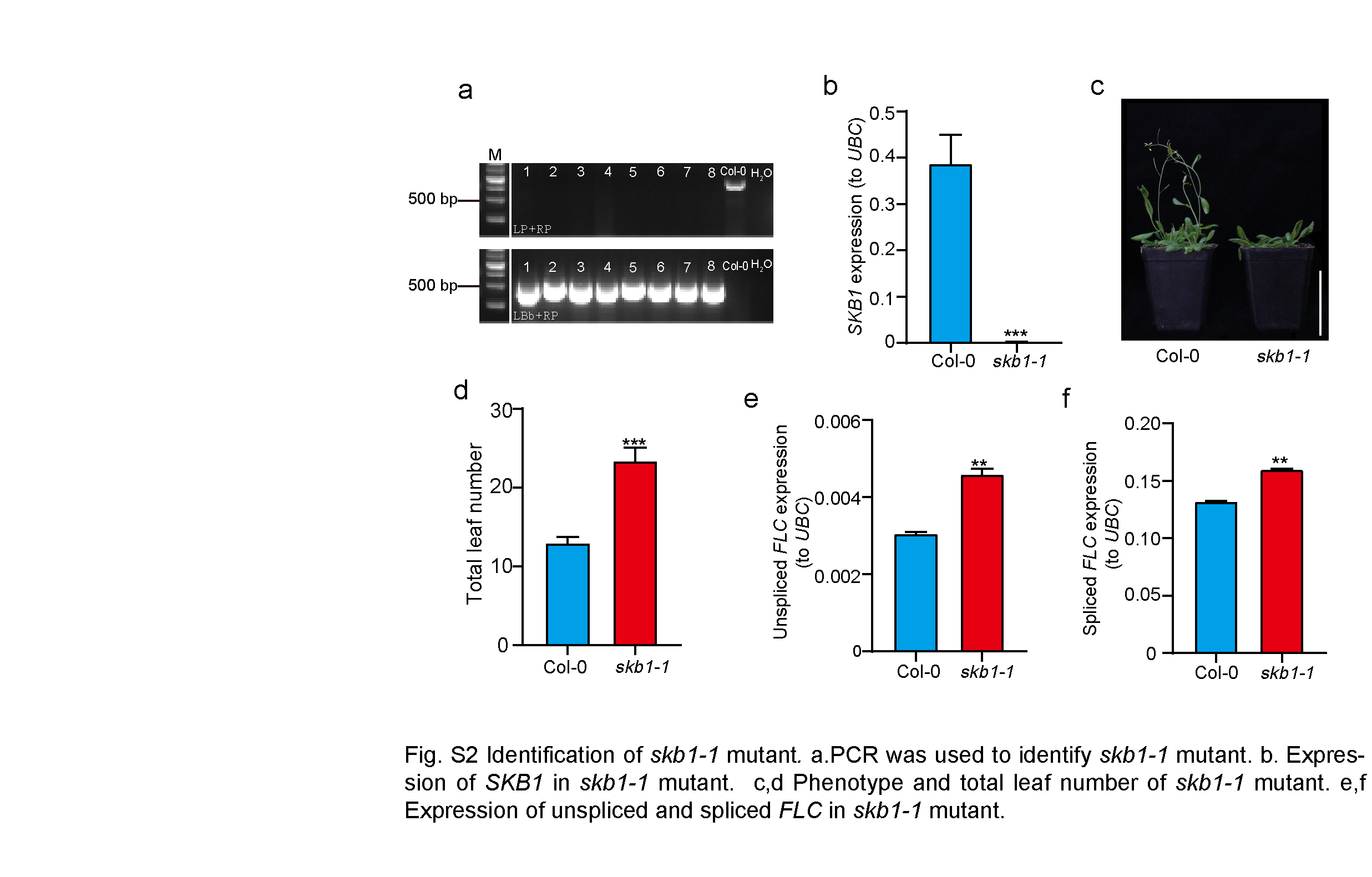

Supplement: Supplementary file 4 — Supplementary Material 4. [file 12870_2024_4887_MOESM4_ESM.tif]

## Top 20 of Pathway Enrichment

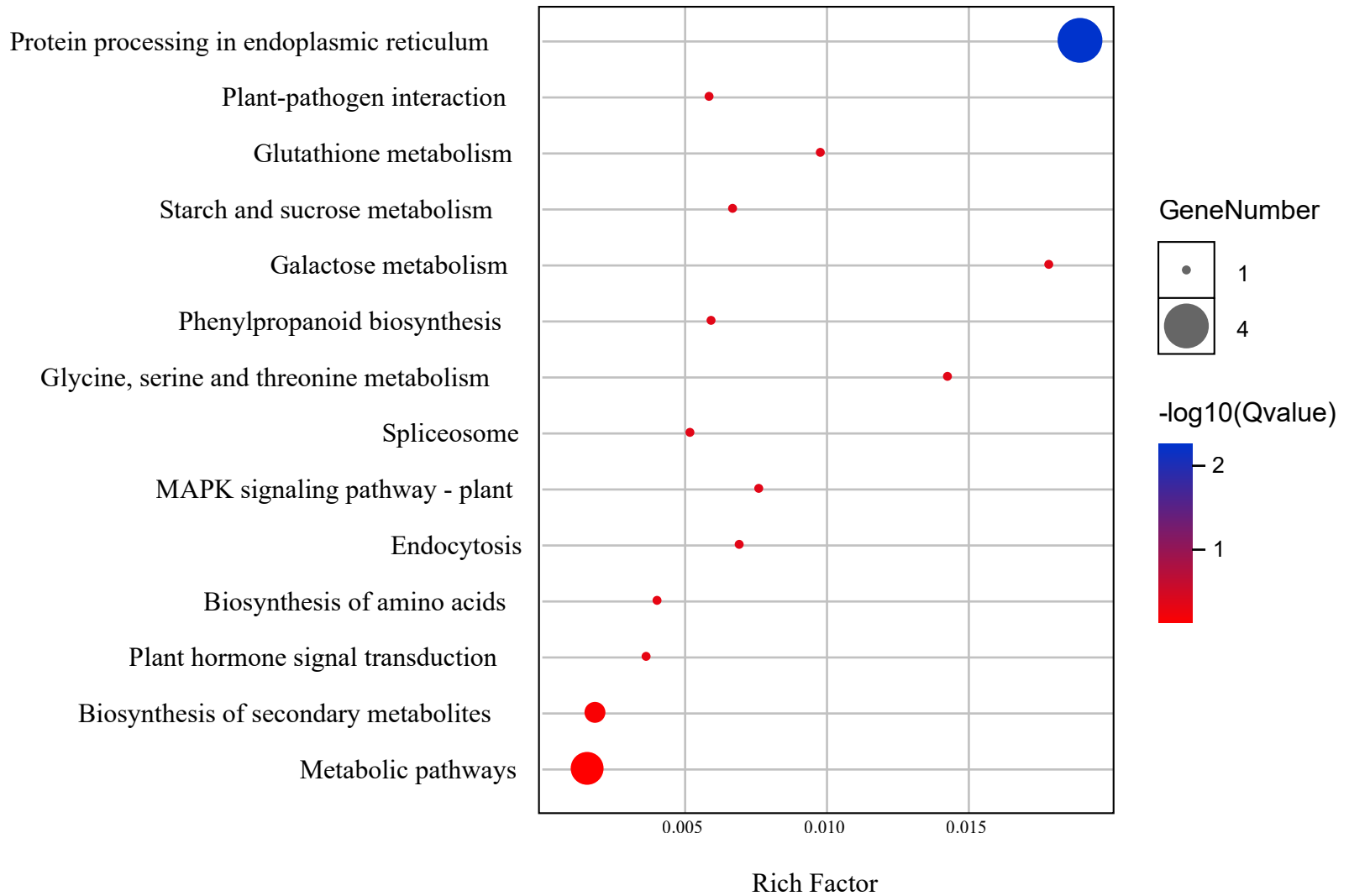

**Figure S4** The KEGG enrichment analysis of 44 common DEGs

Supplement: Supplementary file 6 — Supplementary Material 6. [file 12870_2024_4887_MOESM6_ESM.pdf]
