# Supplementary material for: SISTER OF FCA physically associates with SKB1 to regulate flowering time in Arabidopsis thaliana
Source: BMC Plant Biol. 2024 Mar 15;24:188. doi: 10.1186/s12870-024-04887-y (PMC10941358; doi:10.1186/s12870-024-04887-y)
Supplement: Supplementary file 5 — Supplementary Material 5. [file 12870_2024_4887_MOESM5_ESM.pdf]

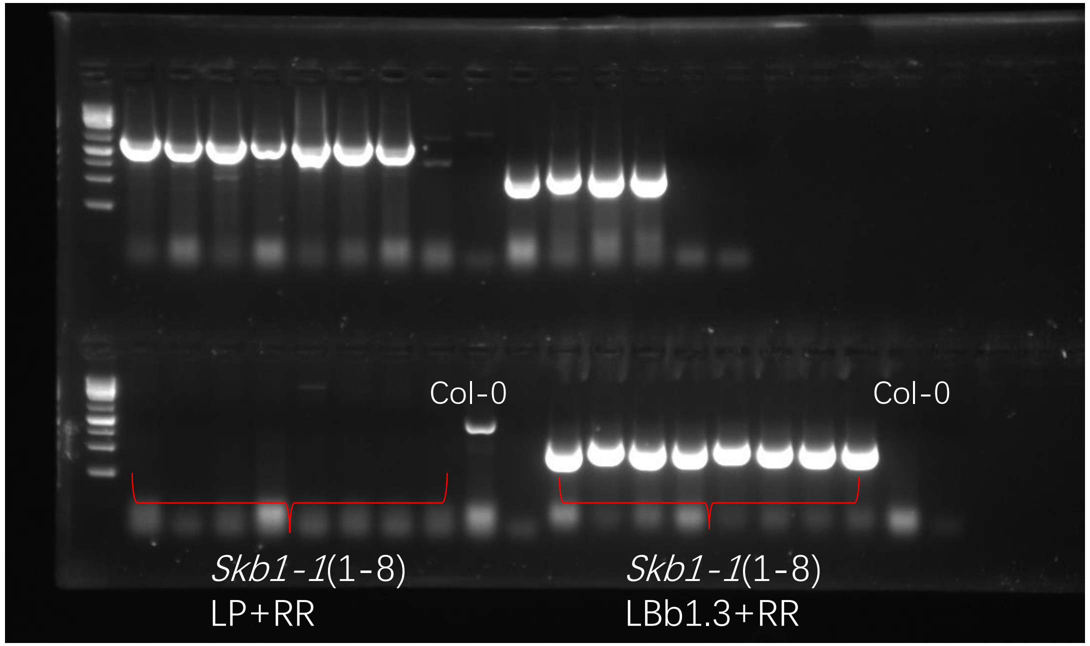

Supplementary Figure S3 The full uncropped Gels and Blots image of PCR identification of *skb1-1* mutant. 20-days *skb1-1* mutant were identified with LP + RP and LBb1.3 + RP primers (n=8). Col-0 as a control.
