# Supplementary material for: SISTER OF FCA physically associates with SKB1 to regulate flowering time in Arabidopsis thaliana
Source: BMC Plant Biol. 2024 Mar 15;24:188. doi: 10.1186/s12870-024-04887-y (PMC10941358; doi:10.1186/s12870-024-04887-y)
Supplement: Supplementary file 7 — Supplementary Material 7. [file 12870_2024_4887_MOESM7_ESM.pdf]

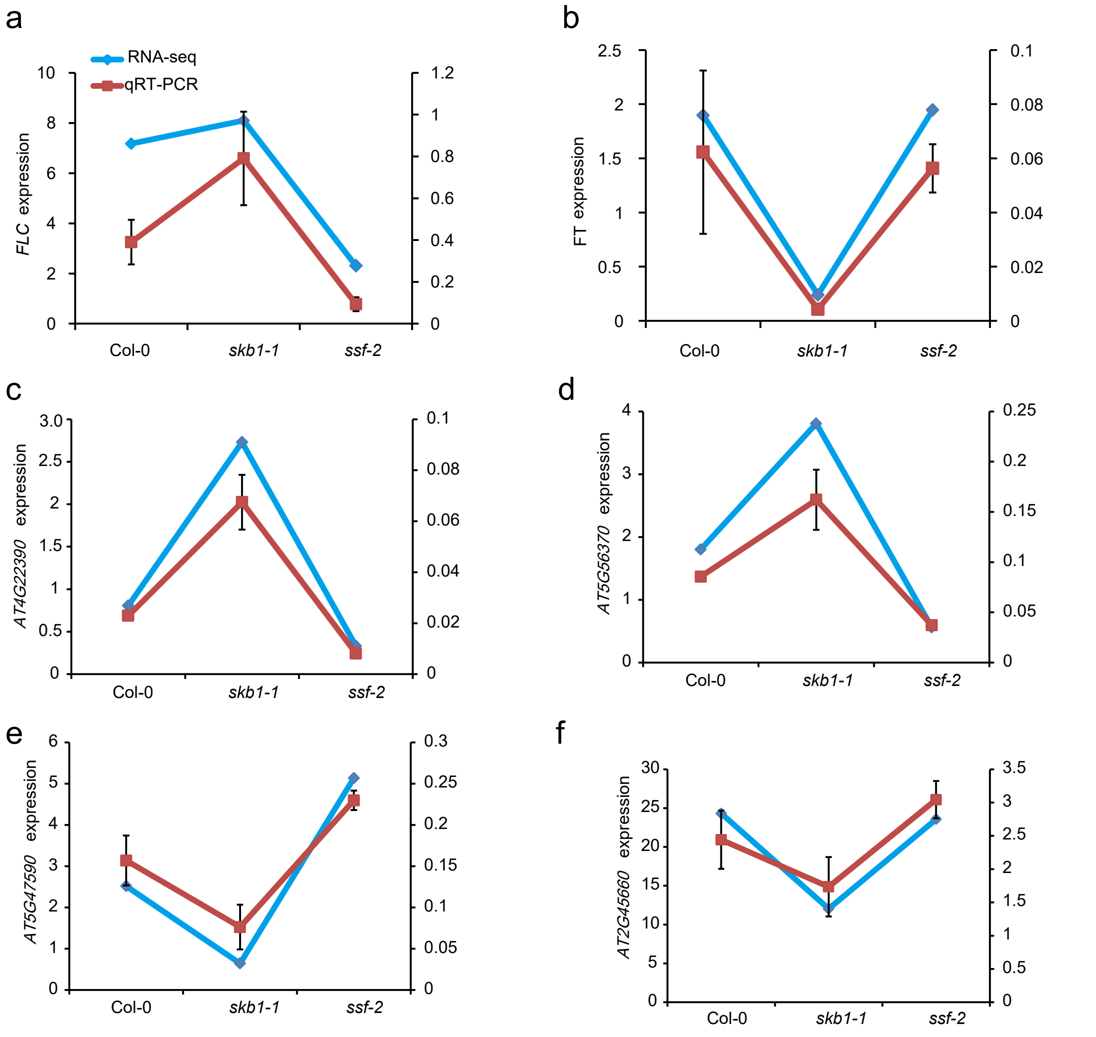

Figure S5 Comparison of qRT-PCR and RNA-Seq results for the selected DEGs. Data shown are the mean values ±SD.
