# Supplementary material for: SISTER OF FCA physically associates with SKB1 to regulate flowering time in Arabidopsis thaliana
Source: BMC Plant Biol. 2024 Mar 15;24:188. doi: 10.1186/s12870-024-04887-y (PMC10941358; doi:10.1186/s12870-024-04887-y)
Supplement: Supplementary file 8 — Supplementary Material 8. [file 12870_2024_4887_MOESM8_ESM.pdf]

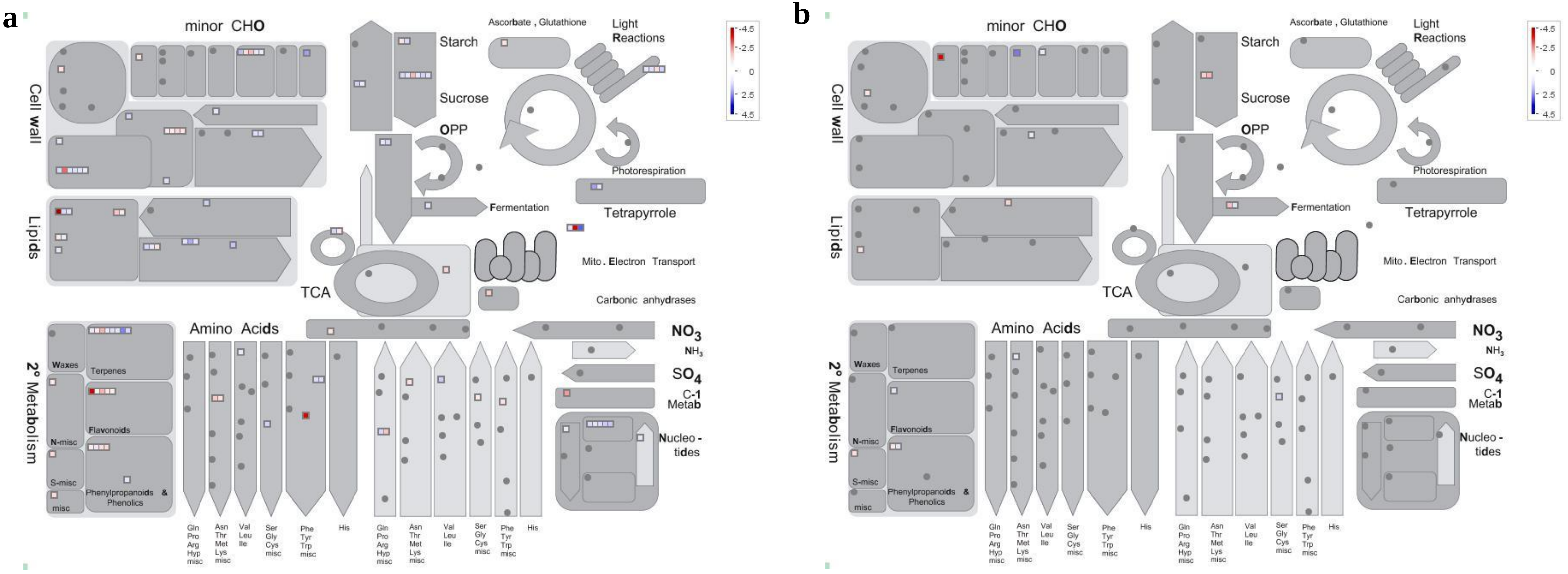

Supplementary Figure S6 MAPMAN analysis of the DEGs between *skb1-1* vs Col-0 (a) and *ssf-2* vs Col-0 (b).
